# Detection of *Ehrlichia ruminantium* infection in cattle in Cameroon

**DOI:** 10.1186/s13104-018-3479-2

**Published:** 2018-06-14

**Authors:** Seraphine N. Esemu, Roland N. Ndip, Lucy M. Ndip

**Affiliations:** 1 Laboratory for Emerging Infectious Diseases, University of Buea, P. O. Box 63, Buea, Cameroon; 2 Department of Microbiology and Parasitology, University of Buea, P. O. Box 63, Buea, Cameroon; 3 Department of Biochemistry and Microbiology, Microbial Pathogenicity and Molecular Epidemiology Research Group, Faculty of Science and Agriculture, University of Fort Hare, Alice, South Africa; 4 Center for Tropical Diseases, University of Texas Medical Branch, Galveston, USA

**Keywords:** *Ehrlichia ruminantium*, Heartwater, Cattle, Polymerase chain reaction, Cameroon

## Abstract

**Objectives:**

*Ehrlichia ruminantium* infection (heartwater) is a major constraint that impacts negatively on the cattle industry development in sub-Saharan Africa and so far, little is known of the presence of heartwater in cattle in Cameroon. This study sought to investigate the prevalence of *E. ruminantium* infection in cattle in Cameroon and to determine the predictors of infection.

**Results:**

A species-specific semi-nested pCS20 polymerase chain reaction was used to screen the buffy coats from 182 cattle (comprising 82 cattle that received intensive tick control regimen and 100 cattle on strategic tick control) from two study sites in Cameroon for *E. ruminantium* DNA in a cross-sectional study. *E. ruminantium* infection was confirmed in 12 (6.6%) of the 182 cattle comprising 11 that received intensive tick control and one on strategic tick control. Of the 12 cattle detected, 11 were apparently healthy and one was clinically diagnosed of heartwater. All DNA sequences of pCS20 amplicons were identical to each other (a representative sequence deposited in GenBank under accession number JQ039939). These findings which have veterinary and epidemiological significance, suggest the need for further investigation to determine the extent and role of heartwater in cattle in Cameroon.

## Introduction

*Ehrlichia ruminantium* (ER) is an obligate intracellular, Gram negative, pleomorphic, tick-transmitted bacterial pathogen recognized as an agricultural biothreat [1, 2]. ER infection causes heartwater, a disease of domestic ruminants and one of the most economically important tick-borne diseases in Africa [3]. In West Africa, heartwater is a major constraint that delays the improvement of the ruminant livestock industry thereby compromising food and nutritional security [4, 5].

In Cameroon, heartwater is one of the major disease problems that delay the cattle industry [6, 7]. Since ER is an obligate intracellular bacterium, its detection requires specific equipment. Hence, most of the reports of the presence of heartwater in Cameroon have been based on the microscopic examination of Giemsa stained brain impression smears of grey matter from deceased cattle [7] or the presence of the heartwater vector, *Amblyomma variegatum* tick [7–11]. Other studies have employed serological methods to demonstrate antibodies against ER proteins in small ruminants [11] while ER signatures have been detected in *A. variegatum* ticks using a DNA-based assay [8]. To date, there is no confirmed evidence of ER infection in live cattle in Cameroon. This study sought to fill this knowledge gap and reinforce the existing reports on the presence of ER in Cameroon by performing DNA-based analysis on live cattle blood as well as determining predictors of infection.

To date, the pCS20 assay is regarded as the most sensitive and reliable test to use for ER detection in cattle and ticks [12, 13]. Even in situations of low parasite levels, the nested pCS20 PCR has been successfully used [14]. Using pCS20 nested PCR, ER infection rates increased from 1.7 to 36% [15]. In this study, we applied a semi- nested pCS20 PCR to determine the prevalence of ER infection in cattle in Cameroon as well as the predictors of infection.

## Main text

### Study design, study sites and cattle population

A cross-sectional survey was carried out between February and April 2010. This study involved sampling cattle from two ranches implementing different tick control strategies and management systems. The study sites, Société de Développement et d’Exploitation des Productions Animales (SODEPA) Dumbo ranch (SDR) and upper farms ranch (UFR) have been described previously [8]. Written authorizations were obtained from the ranch managers before samples were taken. A total of 182 cattle (82 cattle from SDR and 100 from UFR) were sampled. Data on age, sex, breed, tick infestation levels and tick control strategies were recorded in order to evaluate the predictors of infection.

### Clinical samples and DNA extraction

Whole blood (2–4 mL) was drawn from each cattle by venipuncture into EDTA coated vacutainer tubes. The buffy coat was harvested from each blood sample after centrifuging the whole blood at 2000×*g* for 15 min and stored at − 20 °C until used. DNA was extracted from 100 µl of each buffy coat using the DNeasy Tissue Kit (Qiagen, Chatsworth, CA, USA) following the manufacturer’s protocol without modification.

### Semi-nested pCS20 PCR amplification and DNA sequencing

To eliminate the risk of contamination that is inherent in nested PCR, the setting up of PCR reactions mixture, addition of DNA templates and PCR amplification were performed in physically separated areas in the laboratory. Three primers, AB128 (5′-ACTAGTAGAAATTGCACAATCTAT-3′), AB129 (5′-TGATAACTTGGTGCGGGAAATCCTT-3′) and ITM130 (5′-TCAATTGCTTAATGAAGCACTAACTCAC-3′), were used to amplify open reading frame (ORF) 2 of the pCS20 region of ER DNA [14]. AB128 was used as internal forward primer, ITM130 as external forward primer and AB129 as internal and external reverse primer. PCR amplification was carried out as described previously [8]. Ultrapure water (Sigma–Aldrich, Gillingham, UK) was used as negative control and ER CMR Buea 20 (GenBank accession number JQ039914) DNA as positive control. DNA amplification was confirmed by separating the PCR amplicons on 1.5% (w/v) high-resolution agarose gel (Fisher Biotech, Australia) stained with SYBR safe DNA gel stain (Invitrogen, USA). The gel was viewed under ultraviolet light and photographed in a gel documentation system (Alliance 4.7 Chemi and Fluo, Uvitec, Cambridge).

DNA sequencing was performed in both directions for maximum data accuracy. All amplified PCR products were sequenced with the same PCR primers (Inqaba Biotec, South Africa). The BLAST program (National Centre for Biotechnology Information, Bethesda, MD) was used to compare pCS20 sequences to determine species and genotype.

### Statistics

Data were considered statistically significant if p < 0.05. Chi square goodness-of-fit test was used to compare the prevalence of ER in the two study sites, evaluate the hypothesis that tick infestation levels differed between the two tick control strategies and sex.

### Results

#### Characteristics of the cattle population

A total of 182 cattle from two study sites were included in the study (Table 1). The total number of males and females were equal (n = 91). A few of the cattle (n = 10) from SDR were clinically diagnosed of heartwater. Clinical signs observed were loss of appetite, high fever (> 40 °C), incoordination and coughing.

**Table 1:** Epidemiological data and prevalence of *E. ruminantium* infection in cattle included in the study

Characteristics	Location		Total/positive/%	*p* value (from Chi square)
	SDR no. examined/positive/%	UFR no. examined/positive/%		
Total	82/11/13.4	100/1/1	182/12/6.6	0.001
Sex				
Female	22/2/9.1	69/1/1.4	91/3/3.3	0.073
Male	60/9/15	31/0/0	91/9/9.9	
Breed				
Gudali (Zebu)	76/10/13.2	100/1/1	176/11/6.3	0.312
Simgoud^a^	6/1/16.7	0/0/0	6/1/16.7	
Clinical status				
Apparently healthy	72/10/13.9	100/1/1	172/11/6.4	0.655
Suspected heartwater	10/1/10	0/0/0	10/1/10	
Age range (months)				
< 9	0/0/0	7/0/0	7/0/0	
9–24	53/8/15.1	45/0/0	98/8/8.2	
> 24	29/3/10.3	48/1/2.1	77/4/5.2	
Tick control				
Intensive	82/11/13.4	0/0/0	82/11/13.4	0.001
Strategic	0/0/0	100/1/1	100/1/1	
Management system				
Semi-intensive	82/11/13.4	0/0/0	82/11/13.4	0.001
Extensive	0/0/0	100/1/1	100/1/1	
Tick infestation	+++	+		
Mean *A. variegatum* count/cattle	2	10		

+++, heavy tick infestation; +, sparse tick infestation; (%), prevalence

*SDR* SODEPA Dumbo ranch, *UFR* upper farms ranch, *ANAPRI* Associazione Nazionale Allevatori Pezzata Rossa Italiana, *UNIUD* Università di Udine, Italiana, *MINEPIA* Ministere de L’Elevage, des Peches et des Industries Animales (MINEPIA), Cameroon

^a^Trade name of SODEPA for a synthetic breed of cattle which SODEPA has obtained in partnership with ANAPRI/UNIUD and MINEPIA

All cattle from UFR (n = 100) had heavy tick infestation, with a mean of 10 adult *A. variegatum* ticks per cattle while cattle from SDR (n = 82) had sparse tick infestation, with a mean of two adult *A. variegatum* ticks per cattle. SDR was implementing an intensive tick control strategy by dipping all cattle in a dip solution (2% Cypermethrine—ERADIK^®^ or Alpha Cypermithrine—ALPHADIP^®^) after every 2 weeks during the dry season and once every week during the rainy season. This tick control strategy was implemented throughout the sample collection period. UFR was implementing a strategic tick control method (periodically treating cattle with a parasiticide by hand spraying) and throughout the sample collection period (February to April 2010), no tick control was done. The management (grazing) system for cattle in UFR was extensive (free ranging cattle). The management system for cattle in SDR was semi-intensive, hence the cattle grazed freely within the rangeland which is enclosed within a fixed perimeter. Five (2.7%) of the cattle were less than 1 year old. The mean age of the cattle was 3.7 years (range 9 months–15 years).

#### Prevalence of ER DNA and predictors of infection

**Fig. 1:**
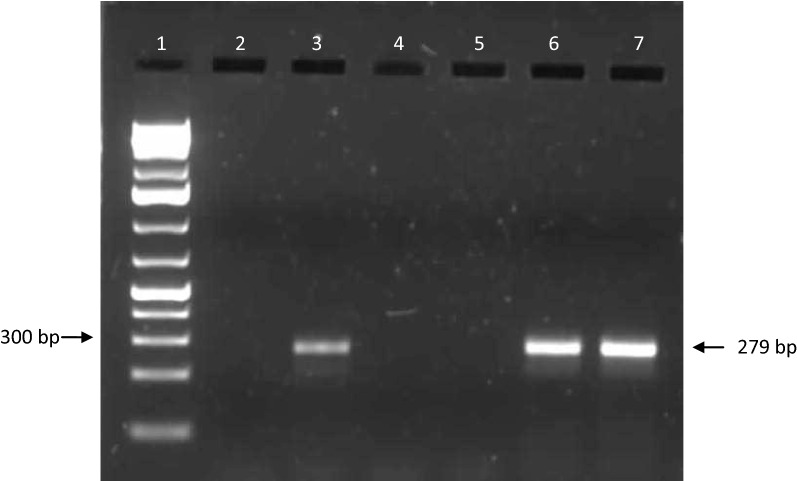
1.5% agarose gel of amplified PCR products from cattle blood. Lane 1: 50–1000 bp molecular weight marker; lane 2: negative control; lane 3: positive control; lanes 6 and 7: positive samples; lanes 4 and 5: negative samples

As expected, DNA amplification produced amplicons of 279 bp (Fig. 1).

The overall prevalence of ER infection in the cattle was 12 (6.6%) of the 182 cattle. Of the 82 cattle from SDR, 11 (13.4%) were positive for ER DNA while one (1.0%) of the 100 cattle from UFR was positive and this difference was statistically significant (χ^2^ = 11.275, p = 0.001, df = 1).

Of the 91 male cattle, 9 (9.9%) had evidence of current ER infection and also three (3.3%) of the 91 cows. There was no statistically significant difference (χ^2^ = 2.418, p > 0.05, df = 1) between prevalence of ER infection in the male and female cattle.

Of the 176 Gudali, 11 (6.3%) were positive for ER infection while one (16.7%) of the six Simgoud was also positive (Table 1). The majority (n = 10, 90.9%) of the 11 Gudali cattle positive for ER DNA came from SDR and 1 (9.1%) from UFR.

Seven (3.8%) of the 182 cattle were less than 9 months old, 98 (53.8%) were within the age range of 9–24 months while 77 (42.3%) were older than 24 months. Eight (66.7%) of the 12 cattle positive for ER DNA were within the age range of 9–24 months while the remaining 4 (33.3%) were older than 24 months. Of the 172 apparently healthy cattle, 11 (6.4%) were positive and 1 (10%) of the 10 cattle clinically diagnosed of heartwater was positive (Table 1).

Cattle that received intensive tick control and maintained under a semi-intensive management system (n = 82, 45.1%) had a higher prevalence (13.4%) of infection. Cattle that received strategic tick control and grazed extensively (n = 100, 54.9%), had a very low prevalence (1%) of infection with ER. Tick control strategy as well as cattle management system are important predictors of ER infection in this study (χ^2^ = 11,275, p = 0.001, df = 1).

#### Processing of PCR amplicons sequences

The sequences of the 12 PCR amplicons were 100% identical to each other, 100% identical to the partial sequence of the pCS20 regions of ER strains CH26 (KX373603) and Hb32 (MG544305) and 99% identical to ER strains Gardel (AY236061), Ball3 (AY236059) and Welgevonden (AY236058). A representative sequence, ER CMR Dumbo 48, was deposited in GenBank under accession number JQ039939.

### Discussion

Heartwater is one of the World Organization for Animal Health’s notifiable diseases [4] because of its economic impact on rural livelihoods in Africa. Our study is the first definitive report of ER infection in live cattle following natural exposure to ER in Cameroon. We confirmed ER infection in 6.6% (12/182) of the cattle. Eleven (91.7%) of the 12 positive cattle were apparently healthy. Previous studies have reported ER in peripheral blood of clinically healthy animals in heartwater endemic areas [16, 17]. Elsewhere, 16 (13.3%) samples positive for ER infection were recorded by pCS20 PCR from blood of 120 cattle that showed no symptoms of heartwater or any other disease [17]. The observation of ER infection in healthy cattle may be an indication of endemic stability or infection by a non pathogenic variant of ER [18].

The difference in ER prevalence in SDR (11/82, 13.4%) and UFR (1/100, 1%) was statistically significant. This difference could be due to the cattle management system and tick control strategy. Although the cattle in SDR grazed within a fixed perimeter, SDR hosts refuge cattle from other areas fleeing their various locations as a result of acute reduction of pasture and/or drinking water during the months of January to April (dry season). The practice of moving livestock from one grazing ground to another may increase their exposure to animal diseases [19]. The type of management system may be a key variable that enhances cattle infection with tick-borne pathogens like ER as this has been reported to determine the host-vector contact time and transmission dynamics [20].

Cattle from UFR had a mean of 10 adult *A. variegatum* ticks per cattle, while those from SDR had a mean of two *A. variegatum* ticks per cattle. Based on this tick infestation rate and the tick control strategies, one would expect a higher ER infection rate in UFR. Interestingly, a very low prevalence of one per cent (n = 100) was observed from UFR. This difference was not statistically significant and the reason for this observation is not clear.

The majority (96.7%) of the cattle studied were Gudali and only six (3.3%) were Simgoud. Simgoud is a synthetic breed of cattle which SODEPA has obtained in partnership with ANAPRI/UNIUD and MINEPIA. The large representation (96.7%) of Gudali in this study is probably due to the popularity of this breed in Cameroon. Gudali make up 65% of total cattle population in Cameroon [6].

Calves between 0 and 9 months have been shown to have a dominant immune response (acquired and innate) to *Anaplasma* and *Ehrlichia* infections and this may be the reason why all the calves in this study were apparently healthy. Adult cattle also benefit from acquired immunity following natural exposure to ER [20]. There was no statistical significance (χ^2^ = 0.77, p > 0.05, df = 1) in the prevalence of ER among cattle of different age groups. Although great diversity has been reported in the *MAP1* gene among ER strains circulating in Cameroon [21], all the ER strains detected in this study were identical to each other in the pCS20 region.

### Conclusions

An ER prevalence of 3.4% was reported in this study with majority of the positive cattle being apparently healthy. Tick control strategy and cattle management system were significantly associated with ER infection. These results suggest the need for further investigation to determine the extent and role of heartwater in the cattle population in Cameroon.

## Limitations

The results from this study cannot be generalized to represent ER prevalence in cattle in Cameroon. This study needs to be expanded to other areas and to other ruminant populations.

## Abbreviations

ER: *Ehrlichia ruminantium*
DNA: deoxyribonucleic acid
PCR: polymerase chian reaction
SODEPA: Société de Développement et d’Exploitation des Productions Animales
SDR: SODEPA Dumbo ranch
UFR: upper farms ranch
